# Computational analysis of receptor tyrosine kinase inhibitors and cancer metabolism: implications for treatment and discovery of potential therapeutic signatures

**DOI:** 10.1186/s12885-019-5804-0

**Published:** 2019-06-17

**Authors:** Jian Li, Kathrin Halfter, Mengying Zhang, Christian Saad, Kai Xu, Bernhard Bauer, Yijiang Huang, Lei Shi, Ulrich R. Mansmann

**Affiliations:** 1 Institute for Medical Informatics, Biometry and Epidemiology, Ludwig-Maximilians-University München, Munich, Germany; 2 German Cancer Consortium (DKTK), Heidelberg, Germany; 3 German Cancer Research Center (DKFZ), Heidelberg, Germany; 4 Department of Computational Science, University of Augsburg, Augsburg, Germany; 5 Department of Orthopaedics, Tongji Hospital, Tongji Medical College, Huazhong University of Science and Technology, Wuhan, People’s Republic of China; 6 Department of Orthopaedics, Physical Medicine and Rehabilitation, University Hospital, LMU, Munich, Germany; 7 Institute of Photomedicine, Shanghai Skin Disease Hospital, Tongji University School of Medicine, Shanghai, People’s Republic of China

**Keywords:** Cancer metabolism, Treatment prediction, Computational modeling, Systems biology

## Abstract

**Background:**

Receptor tyrosine kinase (RTK) inhibitors are frequently used to treat cancers and the results have been mixed, some of these small molecule drugs are highly successful while others show a more modest response. A high number of studies have been conducted to investigate the signaling mechanisms and corresponding therapeutic influence of RTK inhibitors in order to explore the therapeutic potential of RTK inhibitors. However, most of these studies neglected the potential metabolic impact of RTK inhibitors, which could be highly associated with drug efficacy and adverse effects during treatment.

**Methods:**

In order to fill these knowledge gaps and improve the therapeutic utilization of RTK inhibitors a large-scale computational simulation/analysis over multiple types of cancers with the treatment responses of RTK inhibitors was performed. The pharmacological data of all eight RTK inhibitor and gene expression profiles of 479 cell lines from The Cancer Cell Line Encyclopedia were used.

**Results:**

The potential metabolic impact of RTK inhibitors on different types of cancers were analyzed resulting in cancer-specific (breast, liver, pancreas, central nervous system) metabolic signatures. Many of these are in line with results from different independent studies, thereby providing indirect verification of the obtained results.

**Conclusions:**

Our study demonstrates the potential of using a computational approach on signature-based-analysis over multiple cancer types. The results reveal the strength of multiple-cancer analysis over conventional signature-based analysis on a single cancer type.

**Electronic supplementary material:**

The online version of this article (10.1186/s12885-019-5804-0) contains supplementary material, which is available to authorized users.

## Background

Receptor of tyrosine kinases (RTKs) possess highly conserved functional structures from the nematode *Caenorhabditis elegans* to humans and are key components of intracellular signaling pathways such as epidermal growth factor receptor (EGFR), vascular endothelial growth factor receptor (VEGFR), tyrosinkinase (KIT), brain-derived growth factor (BDGF), and others [1]. Therefore, RTKs play an essential regulatory role in diverse, critical cellular processes including proliferation, differentiation, cell survival, apoptosis, and metabolism [2, 3]. Thus, numerous diseases, especially cancer, are highly associated with genetic changes and/or functional abnormalities that result in aberrant activation, pathologic cellular distribution, or dysregulations of RTK [4–7]. Recently, Catalogue of Somatic Mutations in Cancer (COSMIC) provided a substantial volume of mutational information for diverse members of the RTK receptor family for several cancer types. This data inferred that a strong causal link of these receptors to cancer development and treatment must exist [8]. Given these facts, a number of studies have been conducted to investigate and/or develop effective RTK inhibitors. The aim was to improve the therapeutic index and treatment outcome. For instance, imatinib mesylate, a targeted RTK inhibitor, is used to successfully treat chronic myeloid leukaemia as the first line of treatment and treat Gastro-intestinal stromal tumor (GIST) with high risk stratification as the standard of care [9, 10]. Unfortunately, many patients develop a drug resistant disease and relapse due to diverse factors including genetic mutation and molecular mechanisms. Many other RTK inhibitors demonstrated disappointing results in preclinical experiments [11–14]. In order to better understand RTK inhibitor effectiveness and improve their treatment efficacy, studies were conducted to investigate the intracellular signalling mechanisms and complications [15–20]. Unfortunately there are currently few studies that have focused on the possible impact of RTK inhibitors on the cancer metabolism. Even less is known on whether the impact of RTK inhibitor on metabolism is antagonistic or agonistic during or after treatment.

In order to fill these knowledge gaps, we conducted a study to investigate metabolic impact of eight RTK inhibitors on several common types of cancer such as breast, liver, pancreas, central nervous system (CNS), and others. The results of this study may improve our understanding as to how RTK inhibitors can affect metabolism via direct or indirect influence on treatment outcomes or other complications. This in turn may aid in overcoming obstacles in the clinical application of RTK inhibitors, and provide new directions for future studies with the purpose of new therapeutic developments. To our knowledge, this study is the first to investigate the multiple RTK inhibitors’ impact on metabolism of diverse cancers at the molecular level.

## Methods

### The aim, design, and setting of this study

**Fig. 1:**
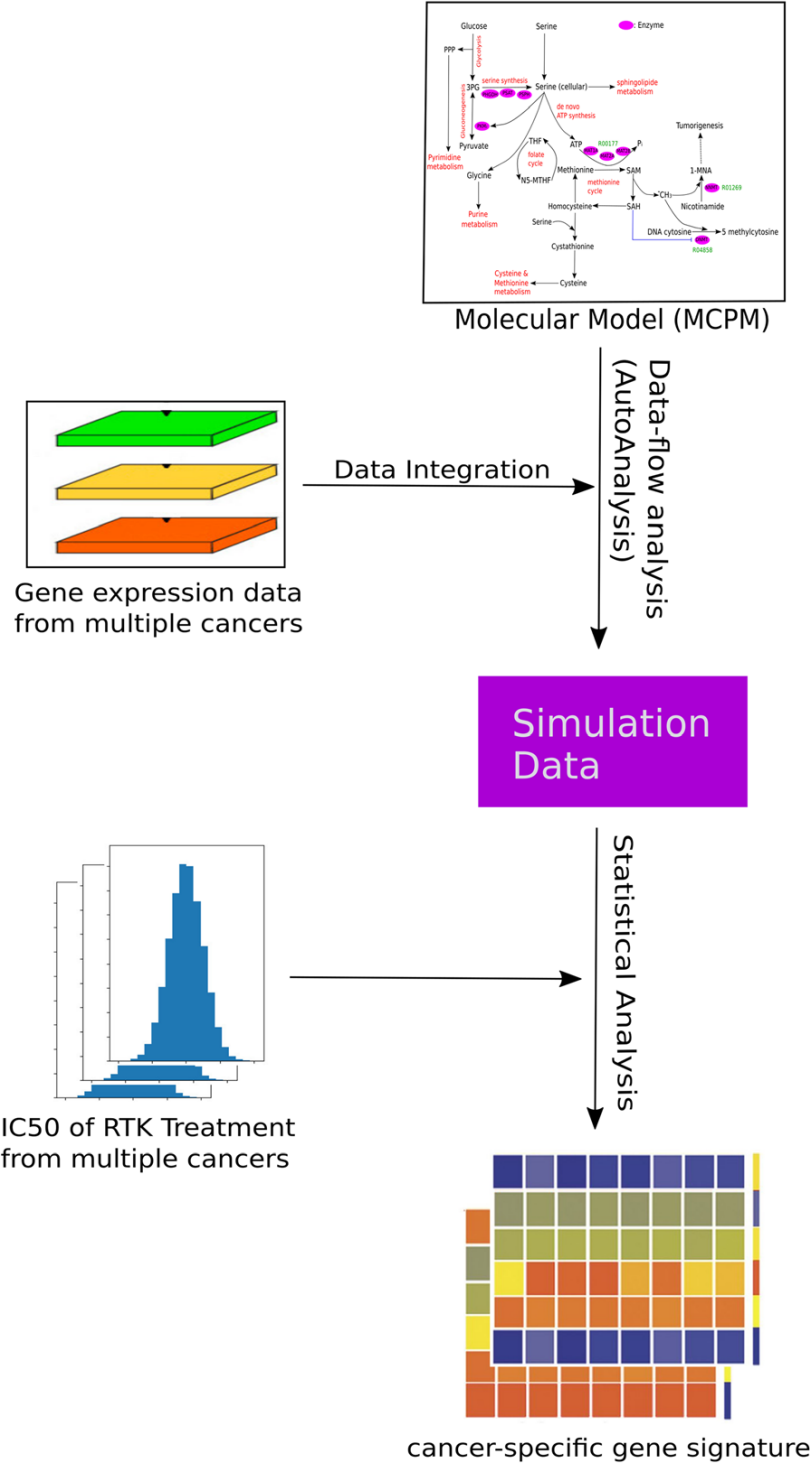
The work flow of this study

The drug sensitivities of eight inhibitors (AEW541, erlotinib, lapatinib, PF-2341066, PHA-665752, sorafenib, TKI258, ZD6474) in different types of cancer were analyzed within the Cancer Cell Line Encyclopedia (CCLE). The aim of this study was to further investigate and characterize the metabolic impact of these eight drugs on these cancer types in order to investigate their impact on cancer metabolism. The CCLE systematically analyzed the drug responses of 479 cancer cell lines derived from 30 cancer types. The measured IC50 values of each of cancer cell lines from CCLE were used to define the response grades of these eight RTK inhibitors’ treatments. We applied the previously published molecular metabolic model, MCMP, to simulate the metabolism of each of these cancer cell lines based on their corresponding GEP from CCLE. The simulation procedure was carried out with the simulation software, AutoAnalyze. Figure 1 visualizes the work flow of this study.

### The molecular metabolic model MCPM and data from CCLE

The published methionine-cycle based metabolic model (MCPM) was manullay constructed based on on information from literatures and the publicly available database, KEGG [21]. The MCPM consists of 3755 components including gene, protein, compound and others [22]. The MCPM has 4750 reactions that are divided into 30 metabolic pathways. In this manner, each model component belongs to one or more corresponding pathways in the model. The crosstalk (overlap) of these pathways is based on literatures and KEGG.

The Cancer Cell Line Encyclopedia (CCLE) published gene expression profiles (GEPs) coupled with pharmacological profiles for 479 cancer cell lines using Affymetrix U133 plus 2.0 array [23]. The data is associated with accession number GSE36139 from Gene Expression Omnibus (GEO) and can be accessed via http://www.broadinstitute.org/ccle.

### Simulation procedure of AutoAnalyze

Each reaction in the MCPM model can be divided into reactants and products. The connection between both is the applied kinetic law. The concentrations of reactants consist of the estimated input data of a reaction, whereas the output data represents the concentrations of products of this reaction. AutoAnalyze uses a data-flow-based calculation approach [24] to compute the concentration of products based on the concentrations of reactants and the corresponding kinetic law with following formula:

1 $$ \mathrm{Output}\left(\mathrm{products},\kern0.5em \mathrm{role}\right)=\Pi\;\left(\mathrm{reactants},\mathrm{role}\right)\ast \mathrm{the}\ \mathrm{kinetic}\ \mathrm{law} $$

The gene expression data from CCLE is the input data for initialization of simulation procedure for AutoAnalyze, which means that only the gene components possess initial values, while the values of other components in the model are initially set to zero. Based on this formula (1), the concentration/value of each of component in the model can be computed and dynamic behavior of pathways can be generated.

### Statistical analysis and metabolic signature generation

We performed spearman correlation between simulated values of each MCPM component and IC50 values of CCLE cancer cell lines for each RTK inhibitor treatment. The results of these correlation analysis were classified for each of these eight RTK inhibitors. The Benferroni correction has been applied for these multiple tests. The MCPM components with spearman correlation > 0.15 has been considered significant. The smallest and common set of significant components of all these RTK inhibitor treatments are defined metabolic signature during this study.

## Results

The simulated values of metabolic components were statistically evaluated to calculate the spearman correlation coefficient with IC50 values of cancer cell lines from CCLE. Table 1 summarizes the significantly affected metabolic components/pathways changed by RTK inhibitor treatment.

**Table 1:** Summary of the impact of eight RTK inhibitors on cancer cell lines from CCLE. For each cancer cell line the metabolism was simulated based on the GEP

Drug	Target	Tumor diagnosis for clinical treatment application (PubMed)	Nr. of Significantly affected Metabolic Components in MCPM model	Nr. of affected metabolic / signaling pathways	Top 5 affected metabolic pathways (Nr. of significantly affected components within the pathway)
AEW541	IGF1R, InsR	Testing phase, none specified (17361225)	125	18 / 2	Purine (19), Glycerolipid (10), Glycolysis (8), Amino-sugar (8), Sucrose (6)
PF-2341066 (Crizotinib)	c-MET, ALK	Non-small cell lung cancer (23724913)	1220	30 / 2	Purine (250), Pyrimidine (114), Inositol-phosphate (62), Fatty-acid (48), Valine/Leucine/Isoleucine (41), Glycolysis (41)
PHA-665752	c-MET	Testing phase, none specified (14612533)	445	24 / 1	Purine (88), Inositol-phosphate (36), Pyrimidine (22), Glycolysis (20), Valine/Leucine/Isoleucine (18), Nicotinate (18)
Sorafenib	ABL, AURKB/C, BRAF, CDK, DDR, EPHA, FGFR, FLT, KIT, MKNK, MAPK, NTRK	Renal cell, hepatocellular, and thyroid cancer (17215530)	1058	30 / 10	Purine (195), Pyrimidine (81), Inositol-phosphate (64), Valine/Leucine/Isoleucine (49), Pyruvate (45)
TKI258 (Dovitinib)	FLT3, KIT, FGFR, VEGFR, InsR, EphA, HER2, IGF1R	Testing phase, none specified (23658459)	1109	28 / 9	Purine (225), Pyrimidine (111), Fatty-acid (59), Inositol-phosphate (53), Glycolysis (47), Pyruvate (47)
Lapatinib	EGFR, ERBB2/4, STK10, PIRK2	Breast cancer (18188694)	504	28 / 7	Purine (82), Pyrimidine (33), Nicotinate (31), Fatty-acid (25), Glycerolipid (22)
ZD6474 (Vandetanib)	EGFR, ERBB2/4, FGFR, ABL, EPHA/B	Thyroid cancer (26678514)	158	27 / 8	Valine/Leucine/Isoleucine (17), Purine (15), Butanoate (11), Pentose (8), Pyrimidine (7)
Erlotinib	EGFR, ABL1, FLT3/4, RET, KIT, RET, SLK, PDGFRA/B, others	Non-small cell lung and pancreatic cancer (21388312)	245	23 / 15	Purine (50), Fatty-acid (12), Nicotinate (12), Glycolysis (11), Fructose (11)

The spearman correlation coefficient was calculated between the simulated values of each component/reaction in the MCPM model and IC50 values of the corresponding cancer cell lines. A spearman correlation coefficient with an absolute value ≥0.15 was considered significant after Benferroni correction

**Fig. 2:**
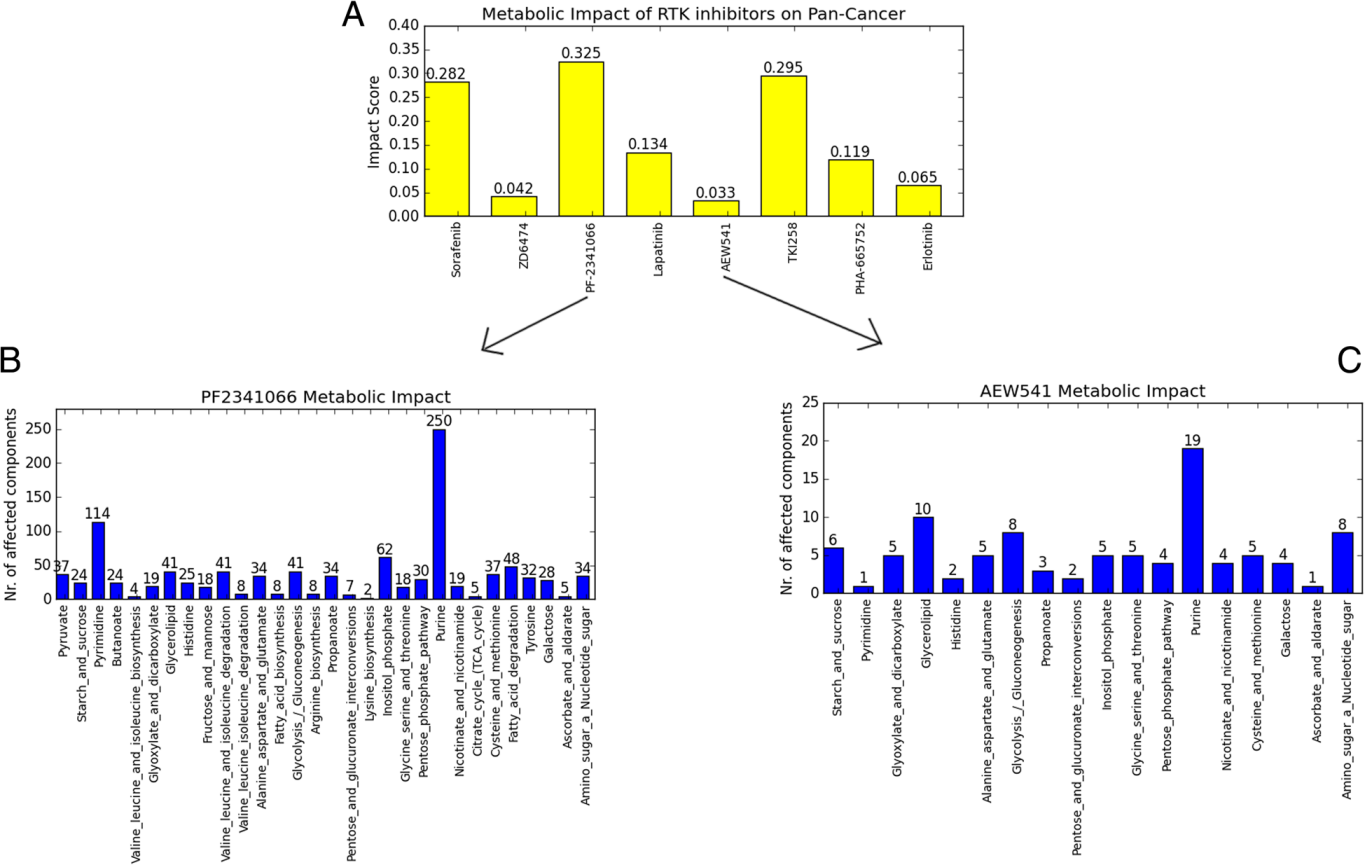
The histogram for the metabolic impact score of 8 RTK inhibitor. **a.** The metabolic impact of drug treatment on multiple cancers from Cancer Cell Line Encyclopedia (CCLE), the impact score is calculated through the division between the number of affected components and the number of all components in the model MCPM; The metabolic impact of PF-2341066 (**b**), AEW541 (**c**) treatment on different metabolic pathways

Based on the simulation results the significantly correlated components/reactions in the model for each of these RTK inhibitors were further investigated to find the highly affected metabolic components / pathways. The aim was to gain further insight into the possible underlying molecular mechanisms of each component / pathway (Table 1). The results show that the purine metabolism pathway is a common metabolic pathway affected by the treatment of all eight RTK inhibitors. Aside from the purine metabolism pathway several other metabolic pathways such as pyrimidine, fatty acid, glycolysis, inositol-phosphate, and valine/leucine/isoleucine were also frequently influenced by the treatment of these RTK inhibitors. According to the number of significantly affected components in the model, the drugs AEW541, ZD6474 and erlotinib had a relative low impact on metabolism, whereas PF-2341066, sorafenib, and TKI258 had relatively strong influence on metabolism (Table 1; Additional file 1). However, the number of drug targets does not correlate with number of significantly affected metabolic components, which indicates that the number of affected signaling pathways and the number of affected metabolic pathways are likewise not correlated. In addition, the resulting levels of influence on metabolism might not directly link to the grade of adverse effects of corresponding drugs. The reason for these different metabolism influences might be the drug target and target binding affinity. For instance, PF-2341066 had the strongest influence on metabolism due to the highest number of significantly affected metabolic components in the model (Table 1). The mechanism of action of PF-2341066 consists of the inhibition of tyrosine-protein kinase Met (c-Met) and anaplastic lymphoma kinase (ALK) with a binding affinity of 11 nM and 24 nM respectively [25]. The strong inhibition of c-Met-dependent and ALK-dependent proliferation, migration, and invasion of tumor cells can directly suppress the metabolic activities of tumor cells. This leads to a clear change of concentration in diverse components in metabolic pathways and results in the strong impact of PF-2341066 on metabolism (Fig. 2a). Among the tested RTK inhibitors, AEW541 had the weakest impact on metabolism as evident by the lowest number of significantly affected metabolic components in the model (Fig. 2b; Table 1). This observation might provide an indication of an overall weak drug efficacy, however, a follow-up study will need to verify this conclusion.

### The metabolic impact and signature of RTK inhibitor in breast cancer

In a refined analysis the focus was placed on the effect of RTK inhibitors on the breast cancer cell lines in CCLE. The simulated values of metabolic components were statistically analyzed to calculate the spearman correlation using the respective IC50 values. The results show that several metabolic pathways such as purine, pyrimidine metabolism pathway in breast cancer cell lines remain affected by the receptor kinase inhibitor (RKI) treatment (Additional file 2). The treatment of AEW541 on breast cancer cell lines resulted in a high impact on all amino acid-related metabolism pathways, which might indicate the potential risk of amino acid metabolism disorder. This disorder could lead to toxic by-products of amino acids that can easily aggregate in blood and urine to cause pathology. However, a follow-up study should verify this side-effect of AEW541 on breast cancer patients. The number of highly affected metabolic components and pathways increased in comparison to the overall results from previous section. This finding might indicate that AEW541 has a higher treatment efficacy in breast cancer compared to the overall efficacy that was observed for all cancer types. The impact on metabolism of PF-2341066, PHA-665752 and TKI258 on breast cancer cell lines was similar to that found for all cancer types. Among these affected metabolic pathways through these three drugs, the inositol-phosphate pathway is often highly affected. This pathway plays an important role in producing signaling molecule IP3, which deeply involves with diverse signaling pathways including PI3K, MET, KIT, VEGFR and others. This result shows how closely metabolism and signaling are connected with each other. However, the impact on metabolism from sorafenib on breast cancer was shifted to sucrose, cysteine/methionine, and fatty acid metabolism. Interestingly, the cysteine/methionine and fatty acid metabolic pathways play pivotal roles in proliferation, migration, and invasion of breast cancer cells [26, 27], while the sucrose pathway provides cancer cells, especially breast cancer, a rich energy resource [28, 29]. This result might provide an interesting clue about the metabolic vulnerability of breast cancer cells. The results of lapatinib, ZD6474, and erlotinib treatment showed that the number of highly affected metabolic components has dramatically increased in comparison to the overall results (Additional file 2). These components are mainly involved in top affected metabolic pathways such as purine, pyrimidine, fatty acid, glycolysis, and others.

**Fig. 3:**
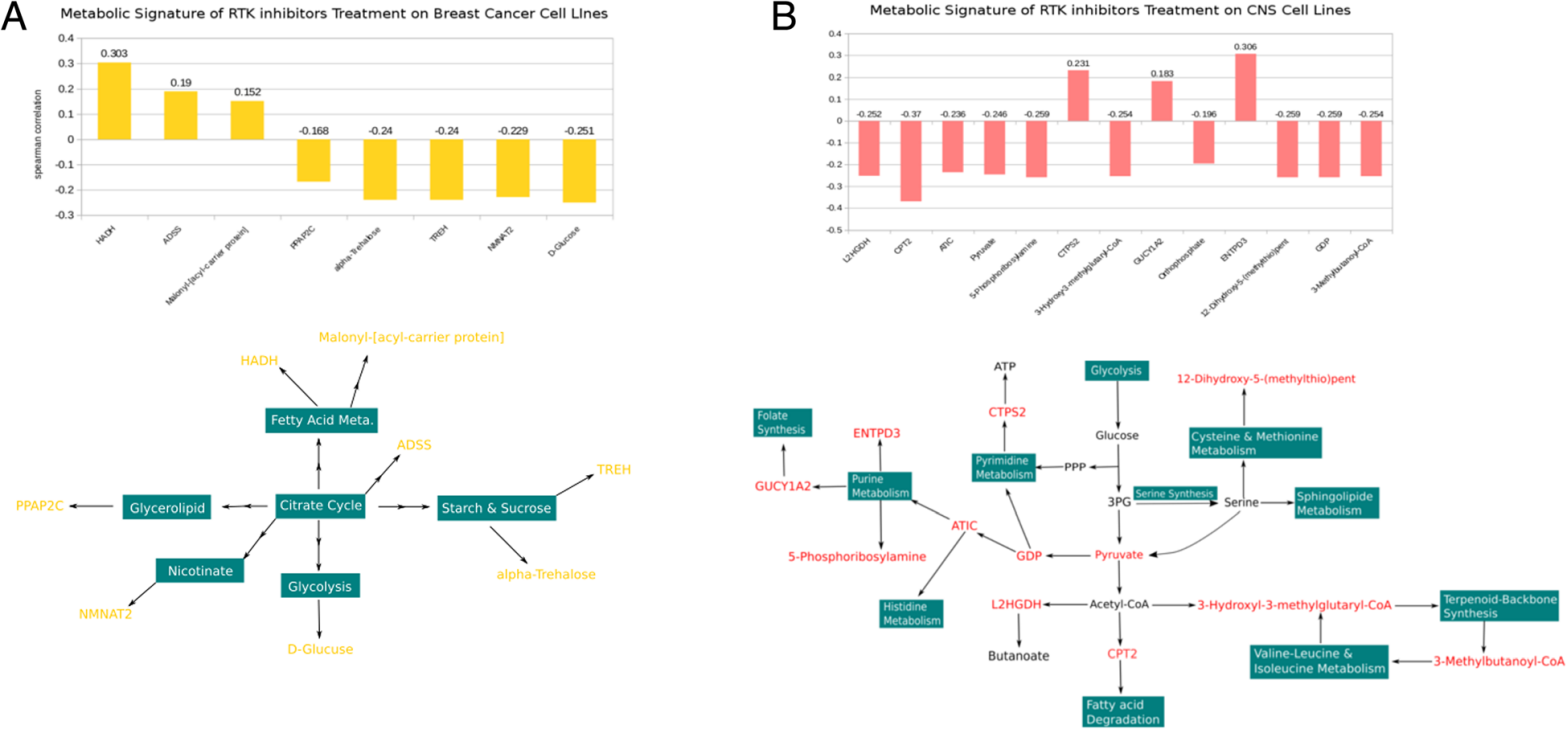
The metabolic signature of RTK inhibitors on cancer cell lines and the corresponding network visualization of relationships between signature components and metabolic pathways. **a**: breast cancer; **b**: CNS

Subsequently, we clustered the highly affected metabolic components from these RTK inhibitors and generated a metabolic signature for breast cancer (MSB) based on the correlation between simulation values of components and IC50 values in order to provide a predictive *in-silico* indication of treatment outcomes of these inhibitors. This metabolic signature consists of eight components from nine different metabolic pathways (Fig. 3a).

### The relationship between metabolic signatures of liver and pancreatic cancer

Liver and pancreas are two essential organs for many metabolic processes in the human body. Both also play an essential role in drug absorption, distribution, metabolism, and excretion (ADME) [30]. Therefore, we wanted to investigate whether a certain relationship or common properties exist between the liver and pancreatic cancer metabolic signatures and RTK inhibitor treatment. Initially a metabolic signature was generated for each of both cancer types, based on the correlation between the simulation values of components and IC50 values on each cancer type. The metabolic signature of liver cancer (MSL) for these RTK inhibtors consisted of 22 components from nine different metabolic pathways, the metabolic signature of pancreas cancer (MSP) for the same RTK inhibitors consists of 16 components from 27 different metabolic pathways (Table 2). These results indicate that these RTK inhibitors might have a stronger metabolic influence in pancreatic cancer than liver cancer. The common metabolic pathways between MSL and MSP follow seven metabolic pathways: fatty acid, glycoglysis, gluconeogenese, citrate cycle, metabolism of diverse sugars including fructose, mannose, sucrose, galactose, pentose, and purine metabolism (Table 2). These pathways are mainly involved with cellular energy supply, conversion, and storage, with a direct link to the physiological functions of both organs. For instance, 14 of 22 components from MSL belong to fatty acid metabolism pathway and all of them have negative correlations with IC50 values of RTK inhibitors. This suggests that a higher effect of an inhibitor may lead to a slower fatty acid metabolism, which indicates that the treatment effect of these RTK inhibitors could severely interfere with the physiological function of liver. Interestingly, the MSP contains Nicotinamid-Adenin-Dinukleotid (NAD) (spearman = − 0.264, *p* < 0.005) and proton (spearman = − 0.185, p < 0.005). This result shows that both metabolites can have negative indicative roles in RTK inhibitor treatment efficacy on pancreatic cancer cell lines. Interestingly, Chini et al. (2014) [31] presented evidence that targeting the NAD metabolism may be a potential novel therapy target for pancreatic tumors by restricting mitochondrial function. Cantó et al. (2015) [32] explained the important role of NAD+ in controlling energy homeostasis to balance cellular interactions between mitochondria and the nucleus, which represents a key control mechanism in the development of several diseases including cancer and neurodegenerative disease. Therefore, we would suggest that MSP could be implicated in functions originating from the mitochondria and nucleus and play a role in RTK inhibitor treatment of pancreatic cancer.

**Table 2:** components of Metabolic signatures for liver- and pancreatic cancer cell lines. The spearman corrections were generated between simulated values of components in the model MCPM and IC50 values of RTK inhibitor treatments on corresponding both cancer cell lines. The Benferroni correction was applied

Metabolic Signatures					
Liver Cancer			Pancreatic Cancer		
Components	Correlation / *p*-value	Pathways	Components	Correlation / p-value	Pathways
trans-Dodec-2-enoyl-[acp]	−0.293 / 2.59E-07	Fatty_acid_metabolism	PDHA1	0.125 / 2.22E-07	Pyruvate_metabolism,Glycolysis_/_Gluconeogenesis,TCA_cycle
SUCLG1	−0.202 / 2.33E-07	Propanoate_metabolism, Citrate_cycle_(TCA_cycle)	CYB5RL	0.244 / 1.68E-07	Amino_Nucleotide_sugar_metabolism
trans-Tetradec-2-enoyl-[acp]	−0.293 / 2.59E-07	Fatty_acid_metabolism	HNMT	0.335 / 2.24E-07	Histidine_metabolism
NAGS	−0.267 / 2.83E-07	Fatty_acid_metabolism	ACOX1	−0.306 / 1.20E-07	Fatty_acid_metabolism,Butanoate_metabolism
(R)-3-Hydroxyhexanoyl-[acp]	−0.328 / 2.02E-07	Fatty_acid_metabolism	TAT	−0.224 / 2.82E-07	Cysteine_and_methionine_metabolism,Tyrosine_metabolism
(3R)-3-Hydroxypalmitoyl-[acyl-carrier protein]	−0.328 / 2.02E-07	Fatty_acid_metabolism	ENTPD5	−0.297 / 1.23E-07	Pyrimidine_metabolism,Purine_metabolism
PFKM	−0.260 / 2.07E-07	Fructose_and_mannose_metabolism,Glycolysis/Gluconeogenesis,Pentose_phosphate_pathway,Galactose_metabolism	NMNAT2	0.256 / 2.06E-07	Nicotinate_and_nicotinamide_metabolism
trans-Hexadec-2-enoyl-[acp]	−0.297 / 2.59E-07	Fatty_acid_metabolism	PCCA	−0.241 / 1.75E-07	Glyoxylate_and_dicarboxylate_metabolism,Valine_leucine_and_isoleucine_degradation
Dodecanoyl-[acyl-carrier protein]	−0.328 / 2.02E-07	Fatty_acid_metabolism	NAT8L	−0.218 / 2.54E-07	Alanine_aspartate_and_glutamate_metabolism
trans-Hex-2-enoyl-[acp]	−0.293 / 2.59E-07	Fatty_acid_metabolism	NAD+	−0.264 / 1.75E-07	28 metabolic pathways
D-Glucose	−0.316 / 1.92E-07	Starch_and_sucrose_metabolism,Galactose_metabolism	H+	−0.185 / 1.56E-07	28 metabolic pathways
Tetradecanoyl-[acp]	−0.328 / 2.02E-07	Fatty_acid_metabolism	Ferrocytochrome b5	0.244 / 1.68E-07	Amino_Nucleotide_sugar_metabolism
NUDT16	−0.324 / 1.99E-07	Purine_metabolism			
(R)-3-Hydroxydodecanoyl-[acp]	−0.328 / 2.02E-07	Fatty_acid_metabolism			
Butyryl-[acp]	−0.328 / 2.02E-07	Fatty_acid_metabolism			
Decanoyl-[acp]	−0.328 / 2.02E-07	Fatty_acid_metabolism			
Octanoyl-[acp]	−0.328 / 2.02E-07	Fatty_acid_metabolism			
(3R)-3-Hydroxytetradecanoyl-[acyl-carrier protein]	−0.328 / 2.02E-07	Fatty_acid_metabolism			
Hexanoyl-[acp]	−0.328 / 2.02E-07	Fatty_acid_metabolism			
2-Deoxyinosine 5-phosphate	−0.330 / 1.89E-07	Purine_metabolism			

The protein N-acetylglutamate synthase (NAGS) (spearman = − 0.267, *p* < 0.005) was identified for the MSL (Table 2). For a healthy liver function this protein can convert toxic ammonium into less toxic urea and is a key enzyme in the urae cycle [33]. Several previous studies showed that a quantitative change in urea cycle enzyme expression directly results in urea cycle disorder and therefore affects other physiological systems including mitochondrial functionality, which can have an influence on the health of patients [34, 35]. A (deoxy)insoine diphosphatase, NUDT16 (spearman = − 0.324, p < 0.005) was identified for the MSL, which shows that this protein has a biomarker-related role in RTK inhibitor treatment on liver cancer cell lines. Interestingly, Iyama et al. (2010) [36] reported that the NUDT16 serves an important hydrolyzation function in the repair processes of nuclear DNA / RNA. The deficiency NUDT16 can induce accumulation of single strand breaks in nuclear DNA, which results in cellular growth arrest. Based on these results, we would suggest that the interesting common property between MSP and MSL are its effect on mitochondrial and nucleous functionality, which might indicate a potential metabolic impact of RTK inhibitor treatment on liver and pancreatic cancer.

### The metabolic impact and signature of RTK inhibitor in central nervous system (CNS)

In recent publications RTK inhibitors have also shown a high potential to be effective in the treatment of CNS-tumor from different stages [37–39]. Thus, we were interested to examine any potentially existing metabolic signature (MSC) in CNS-cancer cell lines from CCLE. The simulated values of the metabolic components from the MCPM and the IC50 values of the CNS-cancer cell lines were analyzed to determine the spearman correlation coefficient and generate the MSC. The MSC consisted of thirteen components from 22 different metabolic pathways (Fig. 3b). Among them are GDP (spearman = − 0.259, *p* < 0.005), pyruvate (spearman = − 0.246, p < 0.005), and orthophosphate (spearman = − 0.196, p < 0.005). The metabolic components that were found are highly associated with the unique metabolic environment of the brain that relies on glucose as its main energy resource. This finding indicates that RTK inhibitor treatment might cause a change in glucose uptake and therefore lead to an inadequate energy supply within the brain. This conclusion may point to a possible adverse effect of the RTK inhibitor treatment. Several independent studies have explained this biomarker-like function of pyruvate for the brain cancer [40, 41], which verifies the important role of pyruvate in MSC indirectly. Further, the gene L-2-hydroxyglutarate dehydrogenase (L2HGDH) (spearman = − 0.252, *p* < 0.005) has been identified in the MSC, showing that the expression level of this gene may be an indication of treatment outcome for these eight RTK inhibitors in CNS-cancer cell lines. A result that is in line with several independent studies [42–44]. Haliloglu et al. (2008) [42] discovered that a mutation in the gene L2HGDH could function as a biomarker based on the results of neuroimaging data from pediatric brain tumors. Vilarinho et al. (2010) [43] explained that the gene L2HGDH serves an important function in cerebrospinal fluid and identified clinical neurophenotypes in different population-cohorts that were associated with the mutation status of this gene. Further, some recent studies provided evidence that pyruvate and L2HGDH might exert influence on the permeability of brain-blood-barrier (BBB) that remains a major obstacle for drug treatment [45, 46]. This result indicates that our proposed MSC might have a certain degree of relationship with BBB and could become relevant in resolving drug delivery challenges across the BBB. The gene ectonucleoside triphosphate diphosphohydrolase 3 (ENTPD3) (spearman = 0.306, p < 0.005) was also identified in the MSC. Interestingly, Peyre et al. (2010) [47] generated a set of biomarkers associated with tumor progression in ependymoma recurrence in children where this gene was also included. This indicates that, even from a different persepective, the role of the gene ENTPD3 in the development of a brain tumor.

### The metabolic impact of RTK inhibitor in lung cancer cell lines

**Fig. 4:**
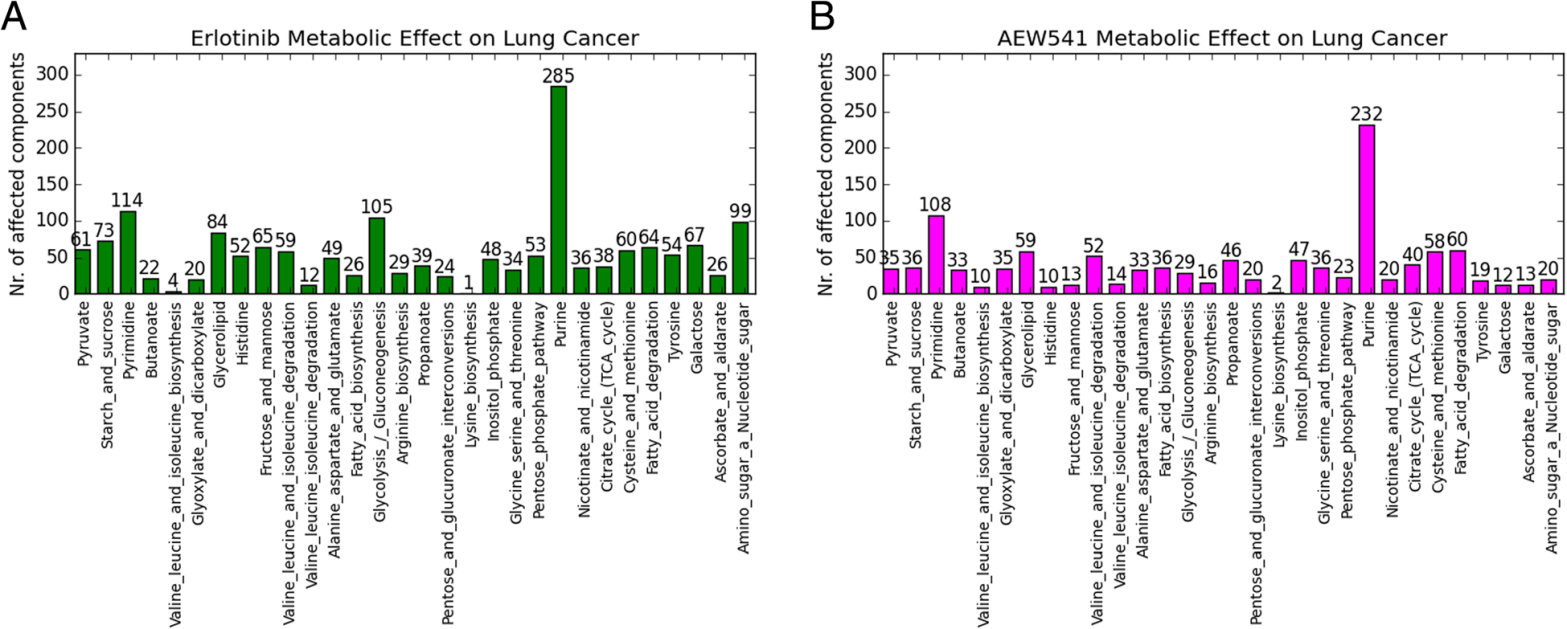
The metabolic effect of drug treatment on lung cancer cell lines. **a**: treatment agent erlotinib; **b**: treatment agent AEW541

Improvement in the clinical outcome of lung cancer could be heavily dependent on the identification of molecular events including metabolism that underlies its tumorigenesis, therefore in this refined analysis the focus was placed on the metabolic effect of RTK inhibitor on lung cancer cell lines in CCLE. The simulated values of metabolic components were statistically analyzed to calculate the spearman correlation with the respective IC50 values from lung cancer cell lines. The result shows that AEW541 and erlotinib have much higher treatment efficacy in lung cancer compared to the overall efficacy that was observed for all cancer types (Fig. 4). Although both drugs do not have a common targetwith AEW541 targeting IGF1R and Insulin-R, whereas erlotinib targets EGFR, KIT, PDGFR, RET among others. However, the metabolic effect of both drugs on lung cancer cell lines do have something in common, for instance, purine- and pyrimidine pathway are highly affected during both treatments. This indicates that inhibition of different signaling pathways could lead to changes of the same metabolic pathways. As Fig. 4a shows, erlotinib treatment in lung cancer cell lines further affects glycolysis/gluconeogenesis and the amino acid, nucleotide, and sugar metabolism. This might explain why the side effect of erlotinib by lung cancer patients can commonly induce clinical symptoms such as poor appetite, nausea and vomiting, fatigue. Moreover, erlotinib treatment also affects the fatty acid metabolism, which may be associated to diarrhea as a treatment side effect. However, not all erlotinib treated lung cancer patients will experience immediate treatment side effects; potentially our observations will be associated to long-term side effects that may become apparent in a follow-up study.

Aside from the purine- and pyrimidine pathway in breast cancer cell lines, the AEW541 treatment further affects glycerolipid, citrate and methionine, fatty acid, propanoate metabolism, which might be linked to the drug side effects (Fig. 4b). The metabolic effect of this drug on lung cancer cell lines was especially elevated compared to the overall efficacy observed for all cancer types. This might indicate that the drug targets of AEW541, namely receptors IGF1R and Insulin receptor, are connected to a vulnerable area within the metabolic pathways active during the development of both cancer types. However, a follow-up study needs to investigate this aspect in detail to determine the key molecular events in lung and breast cancer.

### Computational aspects

In this study, in silico analysis was carried out to simulate the metabolic process for each of the 479 cancer cell lines from CCLE. The simulation was conducted on a standard laptop with a hardware consisting of 2 cores, 2GB RAM, and 8GB Memory. The entire simulation procedure took 20 min and 54 s. The subsequent spearman correlation was repeated a total of 9.301 million times between simulated values of each model component and IC50 values of RTK inhibitor treatment of each CCLE cancer cell lines. This procedure took approximately 27 min.

## Discussion

In this study we utilized the published GEPs from CCLE and integrated these into a published molecular metabolic model, MCPM, through the application of the systemsbiological software AutoAnalyze. The simulation procedure was carried out to generate values of components of MCPM based on the inputed GEP. The simulated values were then statistically analyzed to measure the degree of correlation with the IC50 values of eight RTK inhibitors on cancer cell lines from CCLE. The results show that the purine metabolic pathway is the most affected pathway in the MCPM model by the RTK inhibitor treatment (Table 1). The purines are a group of molecules used by all cells in human body and this metabolic pathway is the major energy carrier and genetic material resources for diverse cellular processes including DNA duplication. Thus, disorders of this pathway are involved in different specific diseases [48, 49]. Given the critical role of this metabolic pathway one should be cautious and consider possible side effects that may occur due to the application of RTK inhibitors and develop treatment strategies to improve the clinical outcomes accordingly. Several other metabolic pathways such as pyrimidine, fatty acid, glycolysis, inositol-phosphate, and valine/leucine/isoleucine were also found to be highly affected by the RTK inhibitor treatment. Since these pathways are associated with several physiological functions our findings strongly support the fact that treatment of RTK inhibitors can have important adverse sides including bone metabolism, linear growth of children, hypertension and glucose metabolism, gonadal function, fetal development among others [50, 51]. These findings thereby justify, at molecular level, several current recommendations that call for prospective thyroid function tests and diabetes managements [52]. Overall, the number of affected signaling pathways did not correlate with the number of affected metabolic pathways during the RTK inhibitor treatments indicating that future studies should put more focus on the crosstalk between metabolic and signaling pathways to fully understand the mechanism and effect of each drug.

The prevalence of breast cancer in our society remains high, which is why we focused one part of our analysis on the effect of RTK inhibitor treatment on breast cancer cell lines from CCLE. The data shows that AEW541 may have a more profound metabolic impact on breast cancer compared to other cancer types. This result is similar to results in recently published independent studies that reported evidence that the insulin-like growth factor 1 receptor (IGF1R) inhibitor, AEW541, reduced proliferation and enhanced the G-1 cell cycle arrest of breast cancer cell lines [53–55]. Another finding from our analysis was the metabolic influence of sorafenib on breast cancer, which is mainly distributed among three metabolic pathways, namely sucrose, cysteine/methionine, and fatty acid metabolism. The physiological functionalities of these metabolic pathways offer an explanation on why sorafenib is generally well tolerated in breast cancer patients [56]. The treatment of lapatinib, ZD6474, and erlotinib on breast cancer cell lines also showed an increased impact on several metabolic pathways such as purine, pyrimidine, fatty acid, glycolysis, and others. This indicates possible adverse effects of these three RTK inhibitors that may occur during their use in the treatment of breast cancer, and might provide an interesting direction for future therapeutic development studies. Different components of the metabolic signature (MSB) for the RTK inhibitor treatments on breast cancer cell lines from CCLE are validated by independent studies for various purposes related to breast cancer. For instance, the protein PPAP2C (spearman = − 0.168, *p* < 0.005) is part of the MSB and has been shown to be highly up-regulated in transformed mesenchymal stem cells in numerous carcinomas including breast cancer [57], which validates the role of this component in the MSB indirectly. The protein hydroxyacyl-CoA dehydrogenase (HADH) (spearman = 0.303, p < 0.005) was also identified in the MSB. Interestingly, Shen et al. (2017) [58] elucidated the important role of HADH in promoting gastric cancer via synergistic effect between fatty acid metabolic pathway and AKT signaling pathway. Therefore, HADH might have a similar function in the development of breast cancer. The protein nicotinamide nucleotide adenylyltransferase (NMNAT2) (spearman = − 0.229, p < 0.005) was identified in the MSB. Sharif et al. (2016) [59] explained that NMNAT2 is a key enzyme in a salvage pathway via p73 for the cancer cell viability, which may verify the important role of NMNAT2 for the RTK inhibitor treatment on breast cancer cells. Further, three components (NMNAT2, glucose, ADSS) from MSB are in line with the result of Lanning et al. (2017) [60] that introduced a metabolic profiling of triple-negative breast cancer cells in order to explain the metabolic vulnerabilities within cancer cell systems. The authors also emphasized the high activity of RTK-related signaling and its possible connection to metabolism thereby verifying our MSB from a different research perspective. A follow-up study could systematically verify the utility of this metabolic signature on breast cancer treatment.

By investigation the characteristics of the metabolic impact on liver and pancreas in regard to RTK inhibitor treatment, we found the commonly affected fatty acid-, glucose-, amino acid-related metabolic pathways. These findings clearly refer to the basic physiological functions of both organs and reveal possible serious implications brought on by RTK inhibitor treatment. Therefore, an appropriate dosing might be essential and critical for RTK inhibitor treatment in managing liver and pancreatic toxicity. To our knowledge, our study could be the first one to apply a computational simulation technique for the investigation of the metabolic properties of two physiologically related organs (liver & pancreas) with cancer status. The involvement of mitochondrial and nucleus functions is common to both metabolic signatures thereby illustrating their therapeutic vulnerabilities during carcinogenesis before metastasis. Although several other studies have published similar findings [36, 32], follow-up studies will need to focus more directly on this issue and perform in-vitro or in-vivo experiments to verify these results.

Due to a large potential for RTK inhibitor treatment on brain tumors a similar analysis as previously described for breast cancer was performed and a corresponding metabolic signature (MSC) was generated as well. Interestingly, we found out that several components from the MSC possess biomarker properties that are relevant for the development and treatment of brain tumors, especially pyruvate and L2HGDH. Both are closely related to the permeability function of the BBB, which presents a major obstacle for any drug treatment targeting any area of the brain. This finding might provide a clear indication for the direction of future studies with the aim of improving brain tumor treatment efficacy. Components from the MSC are distributed among more than 20 different metabolic pathways indicating that RTK inhibitor treatment might have a broad impact on brain metabolism. This might lead to a high risk of adverse reaction during the treatment course. However, follow-up studies should verify this issue through adjustments in the treatment protocol.

For the analysis of this study, we only used the summarized term such as “high impact” on metabolic pathway or “highly affected” metabolic components, but did not further assess or distinguish whether a drug treatment leads to an increase or decrease of metabolic components. The reason for this lies in the numerous and complex crosstalk between signaling and metabolism. From a system level point of view, it is impossible to judge whether an increase of metabolic components could lead to an up-regulation or promotion of other mechanisms or vise verse. Additional and diverse regulation mechanisms within metabolic pathways themselves further adds to its inherent complexity. Therefore only the above mentioned summarized term was taken into consideration. In summary, it is worth mentioning that the number of each type of cancer cell line within CCLE plays a role in the statistical analysis, as dothe number of components defined within each metabolic pathway in MCPM. However, these factors are a common weakness of statistical analysis.

## Conclusion

In summary, our study demonstrated that a computational approach can be applied to conduct a treatment-related molecular signature analysis. This approach is more cost- and time efficient than conventional gene signature-based analysis. Many components from different metabolic signatures computed through our simulation approach are interestingly in line with many independent studies, providing an indirect validation of our approach. Moreover, the analysis scale encompassing multiple-cancer types could yield interesting common properties that would not be possible to be discovered by analysis of only single cancer entity. In future studies, these achieved signatures can be applied in combination with diverse statistical methods including Lasso, ROCR, support vector machine and others for the purpose of pre- and clinical applications.

## Additional files

Additional file 1: The histogram of metabolic effect of eight RTK inhibitors on CCLE. The high number of affected metabolic components indicates strong metabolic effect of corresponding RTK inhibitor in the model MCPM. (DOC 758 kb)

Additional file 2: Summary of the impact of eight RTK inhibitors on breast cancer cell lines from CCLE. (DOC 18 kb)

## Data Availability

The datasets generated and/or analysed during the current study are available in the Gene Expression Omnibus (GEO) with accession number GSE36139 and are also available in the http://www.broadinstitute.org/ccle.

## Abbreviations

ADME: absorption, distribution, metabolism, and excretion
ALK: anaplastic lymphoma kinase
CCLE: cancer cell line encyclopedia
c-Met: tyrosine-protein kinase Met
COSMIC: Catalogue of Somatic Mutations in Cancer
CNS: central nervous system
EGFR: epidermal growth factor receptor
ENTPD3: ectonucleoside triphosphate diphosphohydrolase 3
GEO: Gene Expression Omnibus
GEP: gene expression profiles
GIST: gastro-intestinal stromal tumor
IGF1R: insulin-like growth factor 1 receptor
HADH: hydroxyacyl-CoA dehydrogenase
KIT: Tyrosine-Protein Kinase Kit
L2HGDH: L-2-hydroxyglutarate dehydrogenase
MCPM: methionine-cycle based metabolic model
MSB: metabolic signature for breast cancer
MSC: metabolic signature of cancer
mTOR: the mammalian target of rapamycin
NAD: nicotinamid-adenin-dinukleotid
NAGS: N-acetylglutamate synthase
NMNAT2: nicotinamide nucleotide adenylyltransferase
RKI: receptor kinase inhibitor
RTK: Receptor tyrosine kinase
VEGFR: Vascular Endothelial Growth Factor Receptor
