# Supplementary material for: Computational analysis of receptor tyrosine kinase inhibitors and cancer metabolism: implications for treatment and discovery of potential therapeutic signatures
Source: BMC Cancer. 2019 Jun 17;19:600. doi: 10.1186/s12885-019-5804-0 (PMC6580552; doi:10.1186/s12885-019-5804-0)

**Supplement Information 1**: The histogram of metabolic effect of eight RTK inhibitors on CCLE. The high number of affected metabolic components indicates strong metabolic effect of corresponding RTK inhibitor in the model MCPM.

A.


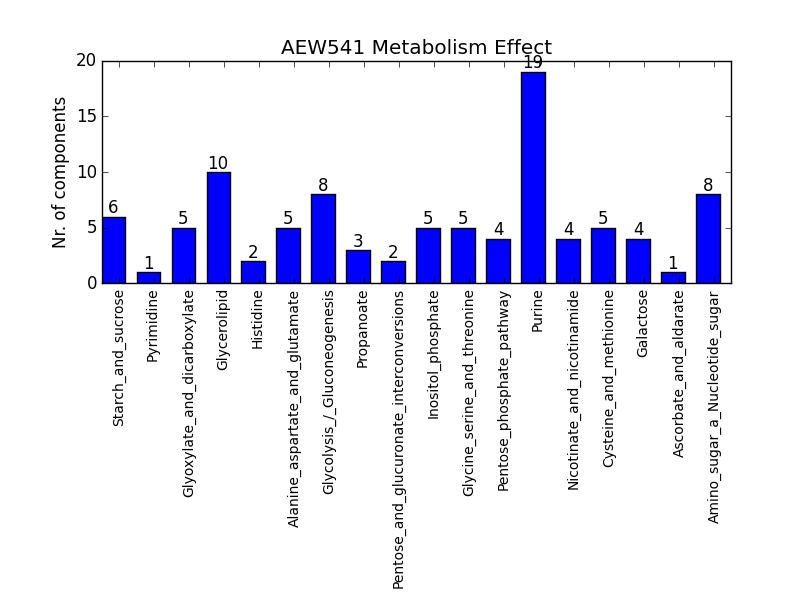


B.


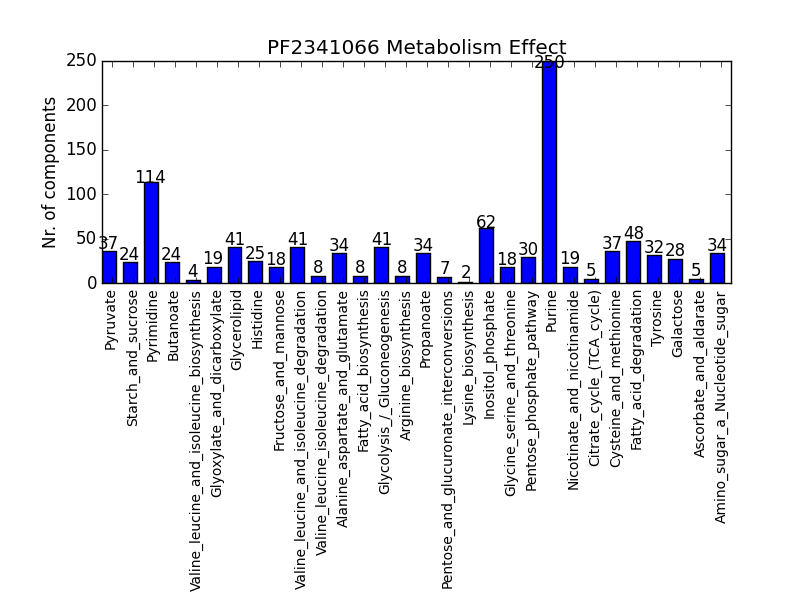


C.


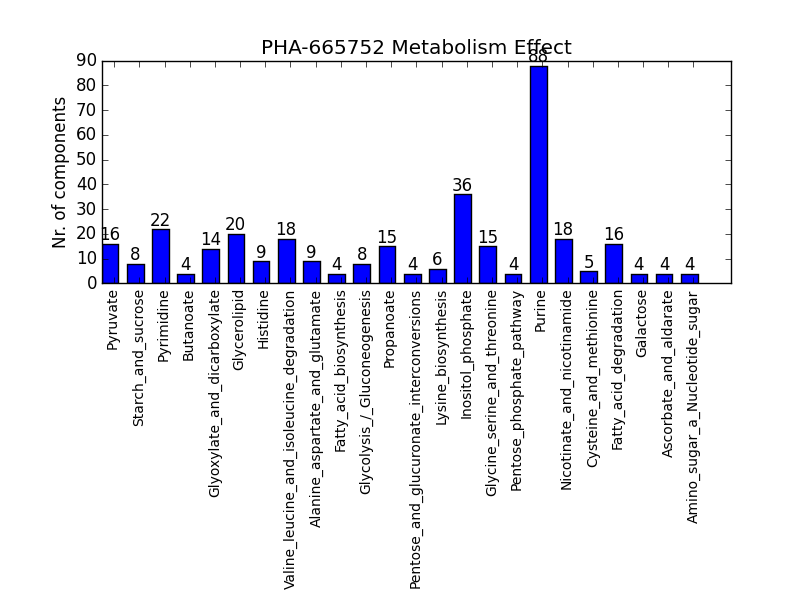


D.


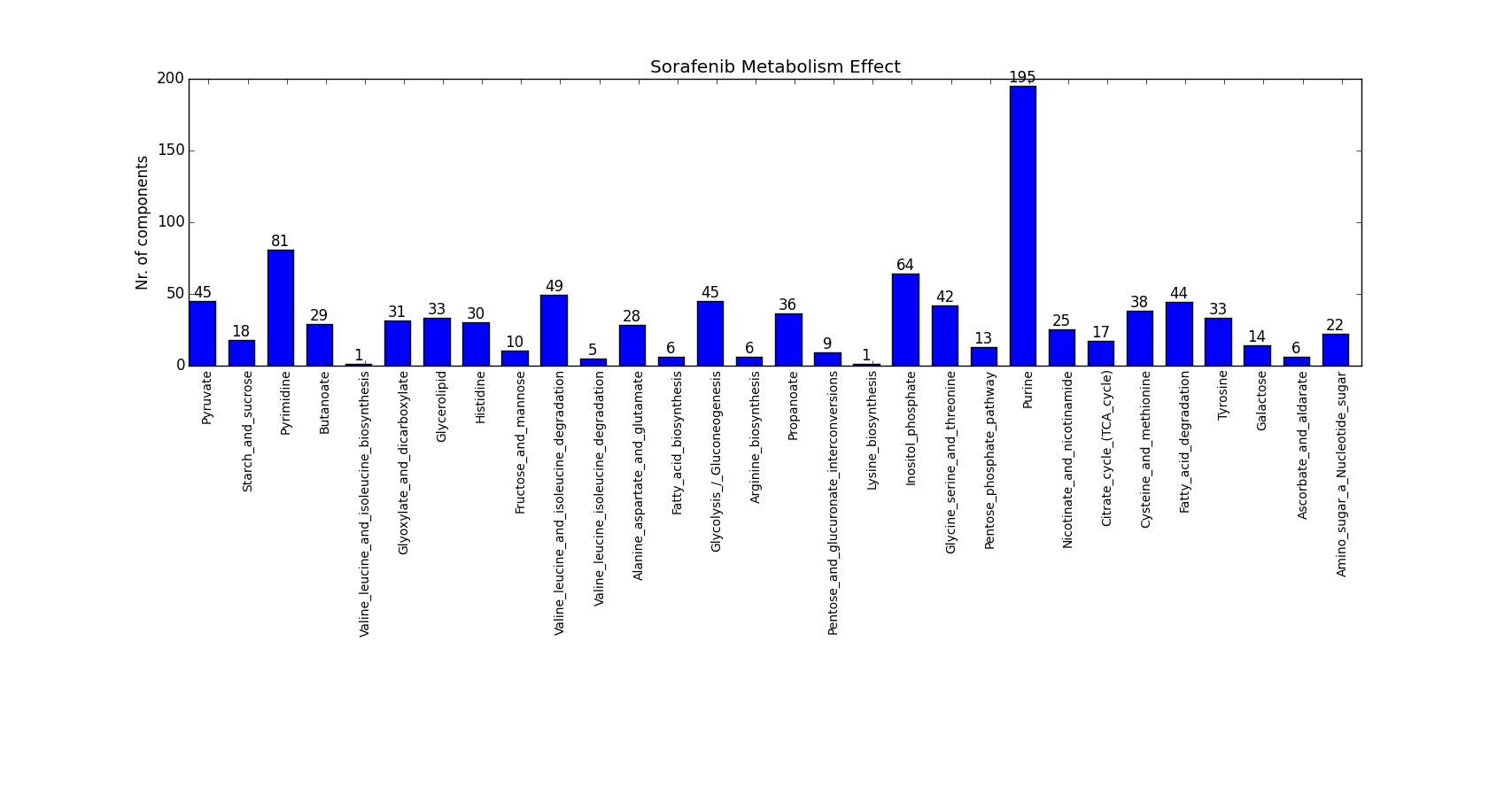


E.


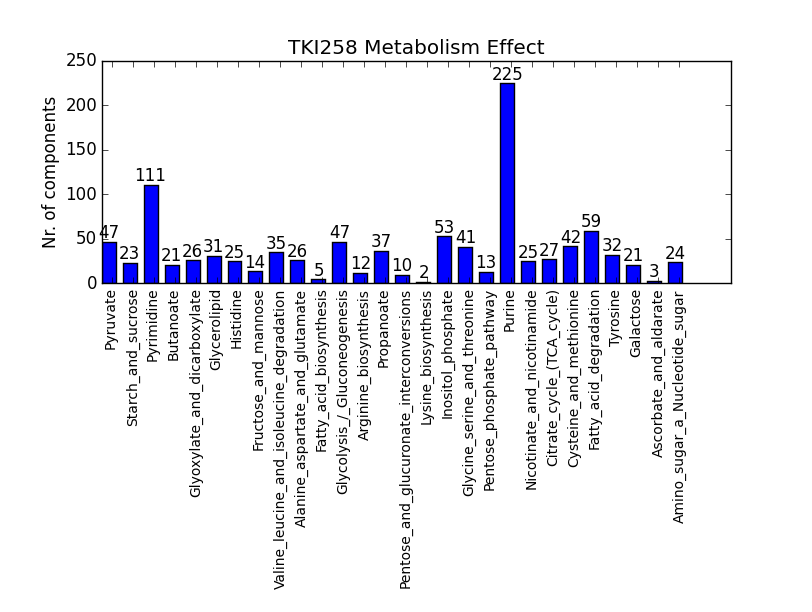


F.


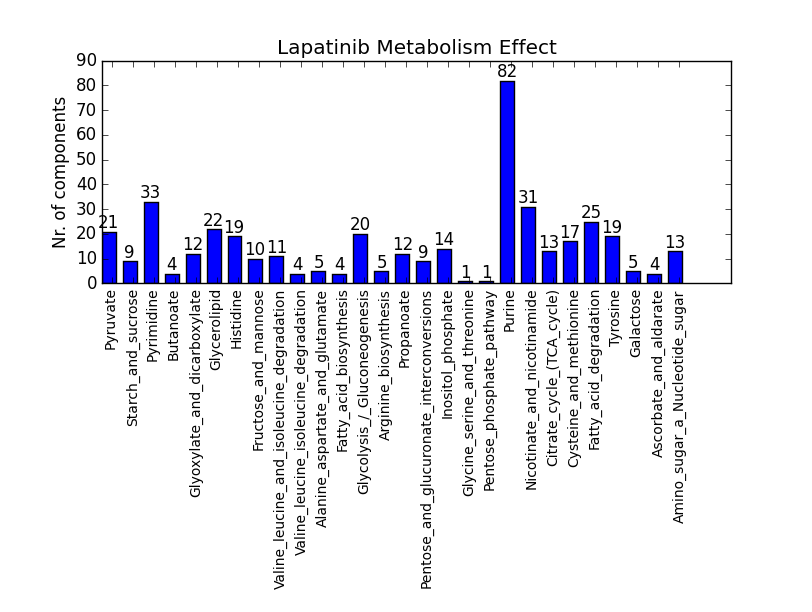


G.


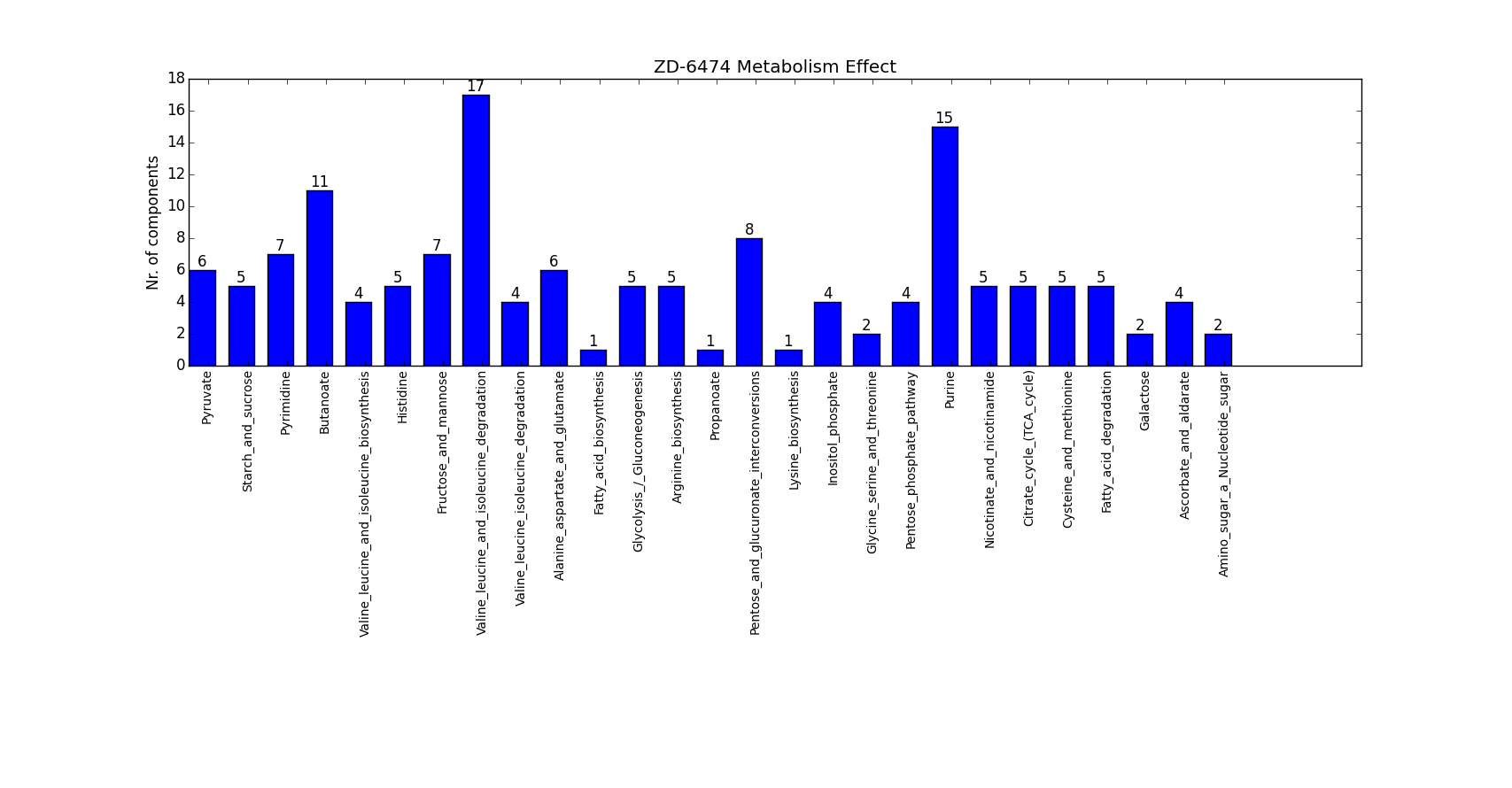


H.


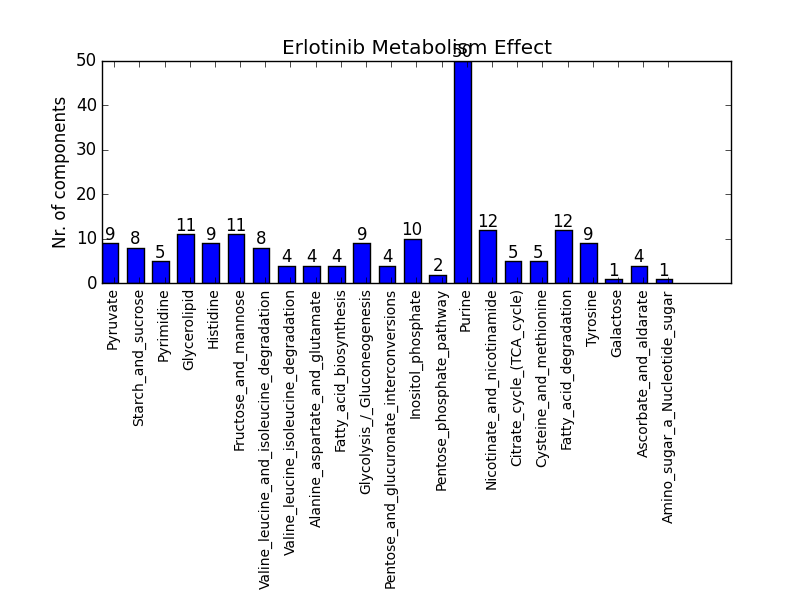

Supplement: Supplementary file 1 — The histogram of metabolic effect of eight RTK inhibitors on CCLE. The high number of affected metabolic components indicates strong metabolic effect of corresponding RTK inhibitor in the model MCPM. (DOC 758 kb) [file 12885_2019_5804_MOESM1_ESM.doc]
