# Supplementary material for: Computational analysis of receptor tyrosine kinase inhibitors and cancer metabolism: implications for treatment and discovery of potential therapeutic signatures
Source: BMC Cancer. 2019 Jun 17;19:600. doi: 10.1186/s12885-019-5804-0 (PMC6580552; doi:10.1186/s12885-019-5804-0)
Supplement: Supplementary file 2 — Summary of the impact of eight RTK inhibitors on breast cancer cell lines from CCLE. (DOC 18 kb) [file 12885_2019_5804_MOESM2_ESM.doc]

**Supplement Information 2**: Summary of the impact of eight RTK inhibitors on breast cancer cell lines from CCLE

| Drug | Target | Nr. of Significantly affected Metabolic Components in MCPM model | Nr. of affected metabolic pathways | Top 5 affected metabolic pathways (Nr. of significantly affected components within the pathway) |
| --- | --- | --- | --- | --- |
| AEW541 | IGF1R, InsR | 617 | 26 | Purine (115), Pyrimidine (50), Inositol-phosphate (31), Glycine/Serine/Threonine (29), Alanin/Asparate/Glutamine (27), |
| PF-2341066 (Crizotinib) | C-MET, ALK | 684 | 30 | Purine (108), Pyrimidine (37), Inositol-phosphate (37), Fatty-acid (37), Valine/Leucine/Isoleucine (39) |
| PHA-665752 | c-MET | 778 | 29 | Purine (91), Inositol-phosphate (63), Pyrimidine (44), Glycolysis (38), Valine/Leucine/Isoleucine (35), Glycerolipid (37) |
| Sorafenib | ABL, AURKB/C, BRAF, CDK, DDR, EPHA, FGFR, FLT, KIT, MKNK, MAPK, NTRK | 725 | 29 | Purine (112), Sucrose (81), Inositol-phosphate (48), Cysteine / Methionine (39), Fatty-acid (34) |
| TKI258 | FLT3, KIT, FGFR, VEGFR, InsR, EphA, HER2, IGF1R | 909 | 30 | Purine (129), Pyrimidine (54), Fatty-acid (45), Inositol-phosphate (62), Glycolysis (49), Valine/Leucine/Isoleucine (55) |
| Lapatinib | EGFR, ERBB2/4, STK10, PIRK2 | 1456 | 30 | Purine (177), Pyrimidine (68), Inositol-phosphate (70), Fatty-acid (61), Glycerolipid (62) |
| ZD6474 | EGFR, ERBB2/4, FGFR, ABL, EPHA/B | 1190 | 29 | Purine (221), Fatty acid (62), Tyrosine (50), Glycolysis (46), Pyrimidine (59) |
| Erlotinib | EGFR, ABL1, FLT3/4, RET, KIT, RET, SLK, PDGFRA/B, others | 995 | 29 | Purine (187), Tyrosine (58), Inositol-phosphate (48), Pyrimindine (57), Glycolysis (43) |
